# Supplementary material for: HIVprotI: an integrated web based platform for prediction and design of HIV proteins inhibitors
Source: J Cheminform. 2018 Mar 9;10:12. doi: 10.1186/s13321-018-0266-y (PMC5845081; doi:10.1186/s13321-018-0266-y)
Supplement: Supplementary file 2 — Additional file 2. Source code of HIVProtI web server. [file 13321_2018_266_MOESM2_ESM.zip › HIVprotI_Source-code/hivprotI/weka-3-6-6/doc/weka/classifiers/meta/FilteredClassifier.html]

FilteredClassifier


---


|  |  |  |
| --- | --- | --- |
| **Overview** \| **Package** \| **Class** \| **Tree** \| **Deprecated** \| **Index** \| **Help** \| **Weka's home** | |  |
| **PREV CLASS**   **NEXT CLASS** | **FRAMES**    **NO FRAMES**     **All Classes** |  |
| SUMMARY: NESTED \| FIELD \| CONSTR \| METHOD | DETAIL: FIELD \| CONSTR \| METHOD |  |


---


## weka.classifiers.meta Class FilteredClassifier

```
java.lang.Object
  weka.classifiers.Classifier
      weka.classifiers.SingleClassifierEnhancer
          weka.classifiers.meta.FilteredClassifier
```

**All Implemented Interfaces:**: java.io.Serializable, java.lang.Cloneable, CapabilitiesHandler, Drawable, OptionHandler, RevisionHandler

---

``` public class FilteredClassifier extends SingleClassifierEnhancer implements Drawable ```

Class for running an arbitrary classifier on data that has been passed through an arbitrary filter. Like the classifier, the structure of the filter is based exclusively on the training data and test instances will be processed by the filter without changing their structure.


Valid options are:

```
 -F <filter specification>
  Full class name of filter to use, followed
  by filter options.
  eg: "weka.filters.unsupervised.attribute.Remove -V -R 1,2"
```

```
 -D
  If set, classifier is run in debug mode and
  may output additional info to the console
```

```
 -W
  Full name of base classifier.
  (default: weka.classifiers.trees.J48)
```

```
 Options specific to classifier weka.classifiers.trees.J48:
```

```
 -U
  Use unpruned tree.
```

```
 -C <pruning confidence>
  Set confidence threshold for pruning.
  (default 0.25)
```

```
 -M <minimum number of instances>
  Set minimum number of instances per leaf.
  (default 2)
```

```
 -R
  Use reduced error pruning.
```

```
 -N <number of folds>
  Set number of folds for reduced error
  pruning. One fold is used as pruning set.
  (default 3)
```

```
 -B
  Use binary splits only.
```

```
 -S
  Don't perform subtree raising.
```

```
 -L
  Do not clean up after the tree has been built.
```

```
 -A
  Laplace smoothing for predicted probabilities.
```

```
 -Q <seed>
  Seed for random data shuffling (default 1).
```

**Version:**
:   $Revision: 1.28 $

**Author:**
:   Len Trigg (trigg@cs.waikato.ac.nz)

**See Also:**: Serialized Form

---

**Field Summary**

**Fields inherited from interface weka.core.Drawable**

`BayesNet, Newick, NOT_DRAWABLE, TREE`


**Constructor Summary**

`FilteredClassifier()`
  
          Default constructor.


| **Method Summary** | |
| --- | --- |
| `void` | `buildClassifier(Instances data)`             Build the classifier on the filtered data. |
| `double[]` | `distributionForInstance(Instance instance)`             Classifies a given instance after filtering. |
| `java.lang.String` | `filterTipText()`             Returns the tip text for this property |
| `Capabilities` | `getCapabilities()`             Returns default capabilities of the classifier. |
| `Filter` | `getFilter()`             Gets the filter used. |
| `java.lang.String[]` | `getOptions()`             Gets the current settings of the Classifier. |
| `java.lang.String` | `getRevision()`             Returns the revision string. |
| `java.lang.String` | `globalInfo()`             Returns a string describing this classifier |
| `java.lang.String` | `graph()`             Returns graph describing the classifier (if possible). |
| `int` | `graphType()`             Returns the type of graph this classifier represents. |
| `java.util.Enumeration` | `listOptions()`             Returns an enumeration describing the available options. |
| `static void` | `main(java.lang.String[] argv)`             Main method for testing this class. |
| `void` | `setFilter(Filter filter)`             Sets the filter |
| `void` | `setOptions(java.lang.String[] options)`             Parses a given list of options. |
| `java.lang.String` | `toString()`             Output a representation of this classifier |

**Methods inherited from class weka.classifiers.SingleClassifierEnhancer**

`classifierTipText, getClassifier, setClassifier`

**Methods inherited from class weka.classifiers.Classifier**

`classifyInstance, debugTipText, forName, getDebug, makeCopies, makeCopy, setDebug`

**Methods inherited from class java.lang.Object**

`equals, getClass, hashCode, notify, notifyAll, wait, wait, wait`

**Constructor Detail**

### FilteredClassifier

```
public FilteredClassifier()
```

:   Default constructor.


**Method Detail**

### globalInfo

```
public java.lang.String globalInfo()
```

:   Returns a string describing this classifier

    :   **Returns:**: a description of the classifier suitable for displaying in the explorer/experimenter gui

---


### graphType

```
public int graphType()
```

:   Returns the type of graph this classifier
    represents.

    :   **Specified by:**: `graphType` in interface `Drawable`
    :   **Returns:**: the graph type of this classifier

---


### graph

```
public java.lang.String graph()
                       throws java.lang.Exception
```

:   Returns graph describing the classifier (if possible).

    :   **Specified by:**: `graph` in interface `Drawable`
    :   **Returns:**: the graph of the classifier in dotty format **Throws:**: `java.lang.Exception` - if the classifier cannot be graphed

---


### listOptions

```
public java.util.Enumeration listOptions()
```

:   Returns an enumeration describing the available options.

    :   **Specified by:**: `listOptions` in interface `OptionHandler` **Overrides:**: `listOptions` in class `SingleClassifierEnhancer`
    :   **Returns:**: an enumeration of all the available options.

---


### setOptions

```
public void setOptions(java.lang.String[] options)
                throws java.lang.Exception
```

:   Parses a given list of options.


    Valid options are:

    ```
     -F <filter specification>
      Full class name of filter to use, followed
      by filter options.
      eg: "weka.filters.unsupervised.attribute.Remove -V -R 1,2"
    ```

    ```
     -D
      If set, classifier is run in debug mode and
      may output additional info to the console
    ```

    ```
     -W
      Full name of base classifier.
      (default: weka.classifiers.trees.J48)
    ```

    ```
     Options specific to classifier weka.classifiers.trees.J48:
    ```

    ```
     -U
      Use unpruned tree.
    ```

    ```
     -C <pruning confidence>
      Set confidence threshold for pruning.
      (default 0.25)
    ```

    ```
     -M <minimum number of instances>
      Set minimum number of instances per leaf.
      (default 2)
    ```

    ```
     -R
      Use reduced error pruning.
    ```

    ```
     -N <number of folds>
      Set number of folds for reduced error
      pruning. One fold is used as pruning set.
      (default 3)
    ```

    ```
     -B
      Use binary splits only.
    ```

    ```
     -S
      Don't perform subtree raising.
    ```

    ```
     -L
      Do not clean up after the tree has been built.
    ```

    ```
     -A
      Laplace smoothing for predicted probabilities.
    ```

    ```
     -Q <seed>
      Seed for random data shuffling (default 1).
    ```

    :   **Specified by:**: `setOptions` in interface `OptionHandler` **Overrides:**: `setOptions` in class `SingleClassifierEnhancer`
    :   **Parameters:**: `options` - the list of options as an array of strings **Throws:**: `java.lang.Exception` - if an option is not supported

---


### getOptions

```
public java.lang.String[] getOptions()
```

:   Gets the current settings of the Classifier.

    :   **Specified by:**: `getOptions` in interface `OptionHandler` **Overrides:**: `getOptions` in class `SingleClassifierEnhancer`
    :   **Returns:**: an array of strings suitable for passing to setOptions

---


### filterTipText

```
public java.lang.String filterTipText()
```

:   Returns the tip text for this property

    :   **Returns:**: tip text for this property suitable for displaying in the explorer/experimenter gui

---


### setFilter

```
public void setFilter(Filter filter)
```

:   Sets the filter

    :   **Parameters:**: `filter` - the filter with all options set.

---


### getFilter

```
public Filter getFilter()
```

:   Gets the filter used.

    :   **Returns:**: the filter

---


### getCapabilities

```
public Capabilities getCapabilities()
```

:   Returns default capabilities of the classifier.

    :   **Specified by:**: `getCapabilities` in interface `CapabilitiesHandler` **Overrides:**: `getCapabilities` in class `SingleClassifierEnhancer`
    :   **Returns:**: the capabilities of this classifier **See Also:**: `Capabilities`

---


### buildClassifier

```
public void buildClassifier(Instances data)
                     throws java.lang.Exception
```

:   Build the classifier on the filtered data.

    :   **Specified by:**: `buildClassifier` in class `Classifier`
    :   **Parameters:**: `data` - the training data **Throws:**: `java.lang.Exception` - if the classifier could not be built successfully

---


### distributionForInstance

```
public double[] distributionForInstance(Instance instance)
                                 throws java.lang.Exception
```

:   Classifies a given instance after filtering.

    :   **Overrides:**: `distributionForInstance` in class `Classifier`
    :   **Parameters:**: `instance` - the instance to be classified **Returns:**: the class distribution for the given instance **Throws:**: `java.lang.Exception` - if instance could not be classified successfully

---


### toString

```
public java.lang.String toString()
```

:   Output a representation of this classifier

    :   **Overrides:**: `toString` in class `java.lang.Object`
    :   **Returns:**: a representation of this classifier

---


### getRevision

```
public java.lang.String getRevision()
```

:   Returns the revision string.

    :   **Specified by:**: `getRevision` in interface `RevisionHandler` **Overrides:**: `getRevision` in class `Classifier`
    :   **Returns:**: the revision

---


### main

```
public static void main(java.lang.String[] argv)
```

:   Main method for testing this class.

    :   **Parameters:**: `argv` - should contain the following arguments: -t training file [-T test file] [-c class index]


---


|  |  |  |
| --- | --- | --- |
| **Overview** \| **Package** \| **Class** \| **Tree** \| **Deprecated** \| **Index** \| **Help** \| **Weka's home** | |  |
| **PREV CLASS**   **NEXT CLASS** | **FRAMES**    **NO FRAMES**     **All Classes** |  |
| SUMMARY: NESTED \| FIELD \| CONSTR \| METHOD | DETAIL: FIELD \| CONSTR \| METHOD |  |


---
